# Adaptive Signal-to-Noise Ratio Indicator for Wearable Bioimpedance Monitoring

**DOI:** 10.3390/s23208532

**Published:** 2023-10-17

**Authors:** Didzis Lapsa, Rims Janeliukstis, Atis Elsts

**Affiliations:** Institute of Electronics and Computer Science (EDI), Dzerbenes 14, LV-1006 Riga, Latvia; didzis.lapsa@edi.lv (D.L.); rims.janeliukstis@edi.lv (R.J.)

**Keywords:** signal quality, bioimpedance, wearable device, continuous wavelet transform

## Abstract

Bioimpedance monitoring is an increasingly important non-invasive technique for assessing physiological parameters such as body composition, hydration levels, heart rate, and breathing. However, sensor signals obtained from real-world experimental conditions invariably contain noise, which can significantly degrade the reliability of the derived quantities. Therefore, it is crucial to evaluate the quality of measured signals to ensure accurate physiological parameter values. In this study, we present a novel wrist-worn wearable device for bioimpedance monitoring, and propose a method for estimating signal quality for sensor signals obtained on the device. The method is based on the continuous wavelet transform of the measured signal, identification of wavelet ridges, and assessment of their energy weighted by the ridge duration. We validate the algorithm using a small-scale experimental study with the wearable device, and explore the effects of variables such as window size and different skin/electrode coupling agents on signal quality and repeatability. In comparison with traditional wavelet-based signal denoising, the proposed method is more adaptive and achieves a comparable signal-to-noise ratio.

## 1. Introduction

Bioimpedance monitoring is a valuable technique to noninvasively assess various physiological parameters such as body composition, hydration levels, heart rate and breathing. However, signals acquired in real-world experimental conditions are often corrupted by noise, posing challenges to the reliability of derived quantities. Therefore, it is essential to evaluate the quality of measured signals to ensure the accuracy of the derived physiological parameter values. This includes applications of bioimpedance signals such as heart-rate monitoring [1], which is the focus of this article. Although heartbeat signals obtained from different individuals tend to be similar, the shapes of individual heartbeat signals are different and variable, and it is important to accurately distinguish between the authentic signal from the interfering noise in terms of heart rate.

Existing approaches for signal quality assessment in bioimpedance monitoring mainly focus on traditional signal-to-noise ratio (SNR) estimation techniques such as autocorrelation [2]. However, these universal methods fail to take into account the specific shape and features of bioimpedance signals; in short, the issue lies in the difficulty of identifying the reference signal that should be compared with the noise. With regard to our application—heart rate detection from the bioimpedance measurements—a problem is that heart rate signals are non-stationary and not normally distributed, as shown further in this paper. Therefore, they are challenging to the existing SNR estimation techniques.

The primary objective of this study is to propose an adaptive signal-to-noise ratio indicator for bioimpedance signals recorded on a wearable device, with the target application of heart-rate monitoring. We focus on signals from the wrist, as it is a typical location for wearable devices. Our contributions are:

- Present a custom wearable device for bioimpedance monitoring.
- Develop a SNR estimation method based on continuous wavelet transform (CWT) to identify wavelet ridges and measure their energy.
- Validate the proposed method using bioimpedance signals obtained in a small-scale experimental trial, with photoplethysmogram (PPG) signals as the reference.
- Compare the performance of the proposed adaptive method with a traditional wavelet-based signal denoising approach.
- Compare the SNR depending on different electrode-to-skin contact materials and other parameters.

The study shows that our adaptive method accurately captures the SNR information. Unlike classical wavelet-based signal denoising and signal-to-noise ratio estimation methods, our adaptive method does not require setting fixed threshold levels or selecting proper threshold types. The PPG signals serve as a reference as they have relatively lower amounts of noise, and as such can validate the separation of bioimpedance signals in “signal” and “noise” components. Developing methods for accurate heart rate estimation based on this decomposition in the signal and noise is in our future plans.

This paper is structured as follows. Section 2 describes the related work. Section 3 describes the experimental platform used in this study. Section 4 explains the novel SNR estimation method and the experimental setup, Section 5 shows the results of this approach, and Section 6 concludes the paper.

## 2. Related Work

Various techniques are commonly employed to denoise biological signals. These include the application of highpass filters [3], lowpass filters [4,5], notch filters [6,7], adaptive filters [8,9], empirical mode decomposition [10,11], blind source separation methods [12] based on the Independent Component Analysis algorithm, and the Fourier decomposition method [13].

Adaptive filters eliminate noise by utilizing a correlated reference signal alongside the original signal. However, their suitability for real-time applications is limited due to the requirement of a reference signal [14]. Nevertheless, the combination of blind source separation methods and adaptive filtering can yield favorable outcomes by generating a reference signal without the need for an implantable sensor [14]. It is important to note, though, that blind source separation methods are computationally intensive, resulting in higher power consumption and processing costs. Consequently, they are not ideal for real-time monitoring.

To address the limitations of the aforementioned techniques and effectively denoise non-stationary signals while preserving the essential signal characteristics, the wavelet transform (WT) methods, namely, discrete wavelet transform [15,16], stationary wavelet transform [14,17] and synchrosqueezed wavelet transform [18] had been used. These approaches operate by decomposing the signals using wavelets and subsequently applying coefficient thresholding to remove noise. These methods have proven to be successful in eliminating the specified types of noise while maintaining the integrity of important signal attributes [14].

Thresholding plays a crucial role in wavelet transform-based signal denoising, as it determines the removal of WT coefficients below the threshold level. Therefore, careful selection of the threshold is essential. The use of the universal threshold rule, proposed by Donoho and Johnstone, has been widely adopted for signal denoising [19,20,21]. However, it should be noted that the universal threshold method is not suitable when the noise variance of the signal is unknown [18].

Attempts to overcome the limitations of conventional thresholding methods were carried out using metaheuristic algorithms. Li et al. [18] use the differential evolution algorithm to dynamically determine the optimal threshold, followed by the application of the soft threshold method to threshold the coefficients obtained from the wavelet transform, similar to the approach adopted in [22].

## 3. Experimental Platform

**Figure 1:**
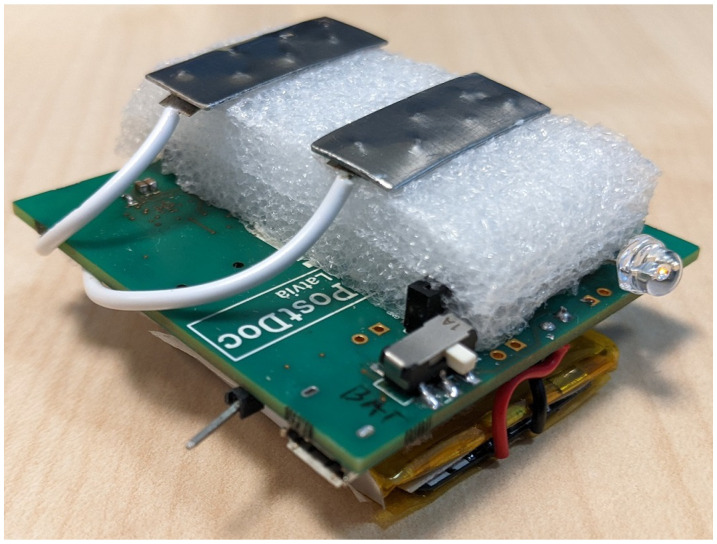
The wearable device used in the experiments, fully assembled view.

As part of the work, we have developed a prototype of a novel wearable device (Figure 1). The main novelty of the device lies in the combination of bioimpedance sensing with a nine-axial inertial measurement unit (IMU) sensor. Coupled with a next-generation, powerful and energy-efficient ARM Cortex-M33 dual-core microcontroller (MCU), this makes it possible to run on-board sensor fusion and activity recognition software [23], making the wearable device suitable for novel applications in health and behavior monitoring.

The main features of the wearable device are

- nRF5340 System-on-Chip (SoC) with dual-core ARM Cortex-M33;
- MAX30001 bioimpedance chip;
- ICM-20994 nine-axial IMU chip, with accelerometer, gyroscope, and magnetometer sensors;
- Li-Ion battery;
- Power sourcing from the USB or from the battery;
- LEDs and a vibration motor for feedback to the user;
- External flash for additional on-board storage;
- User button.

In the short-term future, we aim to develop a new version of the prototype with a smaller form factor. The current PCB size of 53 × 55 mm is meant to enable easy testing and debugging, rather than to be convenient for wearing.

For the experiments described in this paper, we connect two stainless steel electrodes to this wearable device.

### 3.1. Component Selection

#### 3.1.1. Microcontroller and Radio

One of the most important design choices is to find a suitable microcontroller. The latest generation microcontrollers have 32-bit cores and offer much better performance/energy consumption tradeoff than the older 8-bit and 16-bit MCU. Microcontrollers several families were considered, all based on 32-bit ARM Cortex-M cores.

Table 1 and Table 2 show the comparison. EFR32 and nRF5340 have the best overall results across the variety of metrics that are important for our goals: low energy consumption, sufficient RAM and flash memory size, and high performance. From these two, nRF5340 is selected as the one with the better software ecosystem support. While the EFR is the absolute leader in energy efficiency from the MCU considered, it does not have as many existing hardware drivers and experienced programmers as the nRF MCU.

#### 3.1.2. Bioimpedance Measurement Unit

The MAX30001 is a full analog front-end (AFE) solution for wearable applications that includes biopotential and bioimpedance measurements. For bioimpedance input, ECG input, and signal generator output, it features three distinct isolated channels. The biopotential channel detects the electrocardiogram (ECG) waveform, heart rate, and pacemaker edges, whereas the bioimpedance channel measures tissue impedance, such as respiration. ECG and bioimpedance AFE of clinical quality with high-resolution data converter. The 32-word ECG and 8-word bioimpedance first-in first-out buffers (FIFOs) allow the MCU to be turned off for 256 ms while still collecting data. Bioimpedance measurement parameters on the MAX30001 frontend are set as follows: the measurement frequency is 80 kHz, the injected current does not exceed 32 μA, the gain is 80 *v*/*v*, low-pass filter 16 Hz, high-pass filter 7200 Hz, low noise mode selected. High input impedance, low noise, adjustable gain, multiple low-pass and high-pass filter choices, and a high resolution analog-to-digital converter are other features worth mentioning. Critcher et al. [24] provides a far more extensive comparison and examination of this IC, detailing the capabilities supplied by this component.

The existing measurement system uses a bipolar electrode measurement connection, which gives acceptably good results for our requirements. Although the tetrapolar configuration is recognized as more resistant to noise and has a better signal quality [25], the bipolar system is still used for convenience and simplicity [26]. Also, the SNR ratio in the tetrapolar configuration is not always unequivocally better [26].

### 3.2. Wearable Device Design

#### 3.2.1. Design Overview

The wearable device includes the following essential components: nRF5340 central microcontroller, MAX30001 Bioimpedance measurement integrated circuit (IC), and ICM-20948 IMU sensor module.

nRF5340 is a wireless SoC with two ARM Cortex-M33 processors. It supports Bluetooth 5.3, high-speed SPI, QSPI, USB, and an operational temperature of up to 105 °C. The application processor is performance-optimized and may run at 128 or 64 MHz thanks to voltage-frequency scaling. It has 1 MB of flash memory, 512 kb of RAM, a floating-point unit (FPU), an 8 kb 2-way associative cache, and digital signal processing (DSP) instructions. The network processor runs at 64 MHz and is designed to be energy efficient (101 CoreMark/mA). It has a flash memory of 256 kb and a RAM of 64 kb. The SoC supports Bluetooth 5.3 and other protocols including NFC, ANT, IEEE 802.15.4 at the 2.4 GHz frequency band.

The sensor part consists of two data collection channels. The first is the IMU (ICM-209948) Motion Tracking system, which can transmit both the raw data and the results from signal processing on the IMU chip itself. The MAX30001 IC is the second sensor channel. Depending on where it is positioned and what signals are examined, the capacity to measure bioimpedance signals permits the detection of human breathing, heart rate, and other parameters. Both sensors support SPI data transmission, and system interrupts can be used to wake up the main microcontroller from sleep mode.

The device has two power options: micro USB input, and a lithium ion battery. An on-board switch selects the power supply mode. Additionally, 1.8 V voltage regulation IC is included, which then provides the main voltage to all other nodes on the board.

For reading the bioimpedance signals, two stainless steel electrodes are connected to the MAX30001 chip. The considerations behind the electrode design are described in [2].

#### 3.2.2. Energy Consumption

The component selection allows to achieve a long battery life. Using high-accuracy multimeter (Keysight Technologies 34460A, Santa Clara, U.S.) measurements and values from datasheets, we found out that the wearable device with the measurement functionality enabled and Bluetooth Low Energy (BLE) active consumes 3.16 mA average current. When performing bioimpedance measurements without BLE, the current consumption is 2.7 mA. The nRF5340 module consumes approximately 1.5 μA in its sleep mode, while the MAX30001 module consumes 0.6 μA during sleep. During its active operational mode, the MAX30001 module typically consumes 163 μA.

### 3.3. Software

In terms of an operating system for the device, we selected the Zephyr OS, a small-footprint OS designed for use on resource-constrained and embedded systems (https://docs.zephyrproject.org/latest/introduction/index.html (accessed on 14 October 2023)). The main benefits of Zephyr include an extensive ecosystem, *inter alia* direct support from the Nordic Semiconductors company itself, as well as a complete BLE network stack and support for a large number of hardware platforms, including nRF5340-based platforms. Zephyr also features primitives required to work with hardware in a platform-independent way, for example, abstractions for the SPI and I${}^{2}$C protocols, for general purpose input/output (GPIO) and interrupt management, and similar.

## 4. Methods

**Figure 2:**
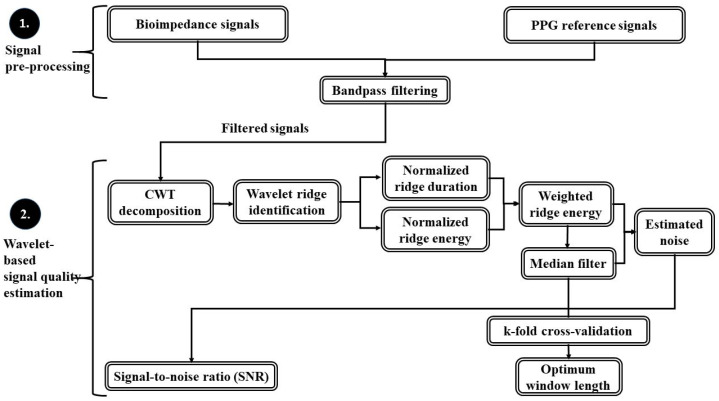
Signal quality estimation in bioimpedance-based human heart rate monitoring applications.

Methodology of the signal quality estimation applicable in human heart beat monitoring is schematically illustrated in Figure 2 and described below.

### 4.1. Band-Pass Filtering

To eliminate undesirable signals such as low-frequency and high-frequency hiss from the data, a standard bandpass filter is employed. The filter’s settings were established based on a frequency response range that was determined using the upper and lower limits of the heart rate, between 30 beats per minute and 300 beats per minute, assuming that these limits were unattainable. The filter is designed using an infinite impulse response (IIR) filter type, as specified by parameter bandpassiir in Matlab filter designer. The frequency range is defined by the following parameters: The lower frequency edge of the first stop-band set to 0.1 Hz, but the lower frequency edge of the pass-band set to 0.5 Hz. The upper frequency edge of the pass-band, set to 5 Hz, but the upper frequency edge of the second stop-band, set to 6 Hz.

To meet the desired frequency response, the filter has specific magnitude constraints, defined by stop-band attenuation. The amount of attenuation in the first stop-band is set to 40 dB and the amount of ripple allowed in the pass-band is set to 1 dB. The amount of attenuation in the second stop-band is set to 50 dB. The filter is designed using the elliptic filter design method. The MatchExactly parameter indicates that the filter design should match the pass-band frequencies exactly. Finally, the sample rate of the signal to be filtered is given as a constant value of 64 samples per second.

### 4.2. Wavelet-Based Signal Quality Estimation

Continuous wavelet transform (CWT) is a time-frequency analysis technique allowing us to observe changes of the signal’s frequency components with time. CWT is performed on the measured signal y(t) to extract the wavelet transform coefficients $W_{y}\left(s,b\right)$ at each scale parameter *s* and time instant *b* according to the formula

(1) $$\begin{array}{ccc} W_{y}\left(s,b\right) & = & \int_{-\infty }^{\infty }y\left(t\right)\times \frac{1}{\sqrt{\left|s\right|}}\times \psi^{*}\left(\frac{t-b}{s}\right)dt \end{array}$$

(2) $$\begin{array}{ccc} & = & \int_{-\infty }^{\infty }y\left(t\right)\times \psi_{s,b}^{*}dt \end{array}$$

where ψ are wavelet functions—special functions satisfying conditions of zero mean and finite energy. Wavelet scale parameter s is related to signal frequency *f* according to this scale-to-frequency conversion

(3) $$f=\frac{\omega_{0}}{2\pi \times s}$$

where $\omega_{0}$ is a central frequency of the wavelet function. CWT decomposition of the measured signal in time and scale (frequency) domains is conveniently viewed through wavelet scalogram (*WS*), which shows proportion of signal energy in time-frequency plane. The highest signal energy concentrates in so-called wavelet ridges, where high energy of a signal corresponds to a specific scale parameter (related to signal frequency) and is traced over the time instants of the signal. It is an instantaneous frequency of the signal. The *WS* is calculated as

(4) $$WS_{ij}=\frac{|W_{y}{\left(s,b\right)|}^{2}}{\sum_{i=1}^{M}\sum_{j=1}^{N}{\left|W_{y}\left(s_{i},b_{j}\right)\right|}^{2}}$$

where i=1,⋯,M is a number of scales and j=1,⋯,N is a number of time instances. The wavelet ridges are identified from two conditions:

1. Derivative of absolute values of the wavelet coefficient with respect to the scale parameter *s* as calculated for all time instants *b* of the signal is zero
  (5) $$\frac{d}{ds}\left|W_{y}\left(s,b\right)\right|=0$$
2. The second derivative of absolute values of the wavelet coefficient with respect to the scale parameter *s* as calculated for all time instants *b* of the signal is negative
  (6) $$\frac{d^{2}}{ds^{2}}\left|W_{y}\left(s,b\right)\right|<0$$

Derivatives of $W_{y}\left(s,b\right)$ of the first and second order with respect to *s* are calculated. For clarity, these quantities are denoted with $D_{1}$ and $D_{2}$, respectively. Time instants and respective scale parameters *s* corresponding to negative values of $D_{2}$ are found and are denoted with $b^{*}$ and $s^{*}$, respectively. The identified wavelet ridges are $W_{y}\left(s,b\right)=W_{y}\left(s^{*},b^{*}\right).$

After the identification of ridges, their duration in number of samples is normalized to the duration of an entire signal.

(7) $$b_{n}=\frac{\sum_{j=1}^{N}b_{j}}{b_{L}}$$

where $b_{L}$ is the duration of signal. The rationale is that the wavelet ridge corresponding to the heartbeat should persist through all of the duration of the signal, while noisy ridges may not have such a stability in time. Then, the energy of each ridge is computed by summing up the wavelet scalogram coefficients corresponding to the ridge scale or along the time axis as $\sum_{j=1}^{N}\left|W_{y}\left(s_{k}^{*},b_{j}^{*}\right)\right|.$

The energy of the wavelet ridge characterizes the intensity of a particular signal component. Next, a normalized energy is calculated for each ridge by dividing the ridge energy by the total energy of the signal, yielding a percentage of energy associated with each wavelet ridge.

(8) $$e_{n}=100\times \frac{\sum_{j=1}^{N}\left|W_{y}\left(s_{k}^{*},b_{j}^{*}\right)\right|}{\sum_{i=1}^{M}\sum_{j=1}^{N}\left|W_{y}\left(s_{i},b_{j}\right)\right|}$$

Afterwards, this normalized energy is weighted by the duration of the wavelet ridge to obtain the weighted ridge energy as

(9) $$e_{w}=e_{n}\times b_{n}$$

The weighted ridge energy takes into account both ridge stability in time and also intensity of the ridge. Hence, the signal component corresponding to heartbeat should have a very high weighted energy compared to other ridges.

**Figure 3:**
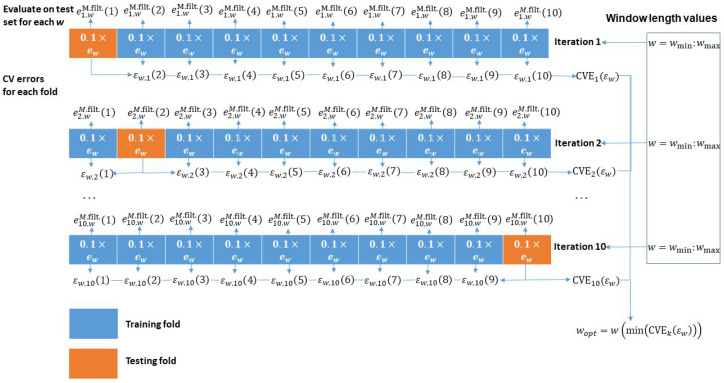
k-fold cross-validation scheme with 10 folds to optimize window length selection for median filtering.

A cornerstone of the proposed signal quality estimation method is estimation of noise content. Here, the median filter is applied to the weighted ridge energies. The choice of median filter instead of other filters is based on the distribution of $e_{w}$ values. As this distribution is not normal, since relatively few samples have high values, median filter is more robust to such outliers compared to, for example, mean filter. Median filter, however, requires us to set a window length in which the median operation is to be performed. The choice of window length is optimized using k-fold cross-validation technique in the following manner: The weighted energy data $e_{w}$ have been split into 10 folds, where 9 folds were used for applying the median filter to the data and obtaining filtered data $e_{w}^{M.filt.}$ at pre-defined window length values. It constitutes a training data set. The remaining one fold was used for testing where a mean-squared error metric between the filtered data and data in this one fold (testing data set) was computed. This operation was repeated by cycling through all 9 folds and the one fold for testing was fixed. The following 9 iterations, however, cycled through the fold for testing so that eventually all 10 folds were used for testing once. This scheme is shown in Figure 3.

When the optimum value of window length has been chosen. The noise component of a signal is determined as a difference between the weighted ridge energy $e_{w}$ and a filtered $e_{w}$ with a median filter as $noise=\sum_{i=1}^{M}{\left|e_{w,i}-e_{w,i}^{M.filt.}\right|}^{2}.$

The proposed signal quality indicator, also called signal-to-noise-ratio (SNR) is defined as

(10) $$SNR=10\times \log_{10}\frac{\sum_{i=1}^{M}e_{w,i}^{2}}{noise}$$

The numerator expresses the total energy of the signal y(t) across all frequencies and time instants, whereas the denominator reflects the part of the signal related to noise—information-carrying signal components subtracted from the total signal. Following other definitions of the SNR, its proposed version is also expressed in decibels.

### 4.3. Experimental Measurements

**Figure 4:**
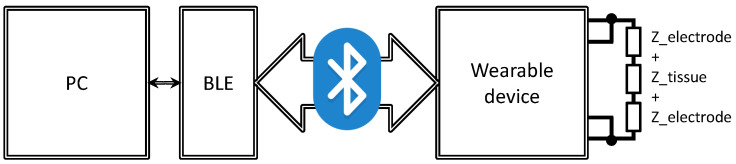
Bioimpedance wearable device equivalent circuit connected to a computer via Bluetooth.

The experiment was carried out with the wearable device shown in Figure 1 and described in Section 3, Experimental Platform. The complete measurement system also includes a BLE receiver module, a computer as a data receiving unit, as well as an impedance model of organic human tissue as Z tissue, shown in Figure 4. The prototype includes a pair of stainless steel electrodes that are supported by a layer of foam to improve flexibility and reduce pressure on the skin. The dimensions of prototype PCB are 55 mm × 52 mm × 30 mm, including the battery and electrodes. The initial electrode size was approximately 8 mm × 18.5 mm, but this was later changed to larger electrodes with dimensions of 18.5 mm × 44 mm to improve the contact area with the skin. The decision to use stainless steel electrodes was based on previous research on electrode materials and their properties, with particular emphasis on their effectiveness in heart rate detection [2].

The MAX30001 integrated circuit was configured to sample bioimpedance data at a rate of 32 or 64 samples per second. Multiple readings were collected and transmitted to a PC via the nRF5340 using Bluetooth Low Energy (BLE) protocol. A Python script was used to collect data at the user end. The collected data were then transferred to Matlab for analysis, where heart rate was extracted and analyzed over time.

Bioimpedance signatures were measured on nine individuals (subjects I to IX). Informed informed consent was obtained from all experimental subjects. Biological data of the test subjects are shown in Table 3. Each measurement session consisted of simultaneous bioimpedance and PPG measurements, where a PPG sensor was held between the thumb and an index finger. The wearable device was put on the wrist parallel to ulnar and radial arteries. The test subjects were sitting still during the measurement session. The effect of two different couplings between skin and electrodes was explored. A total of 10 measurements were performed using a gel “E.C.G. AND TENS GEL” [27] and 10 measurements were performed using hydrogel sheets “MODEL MA1017” [28] with size 4 × 6 cm, cut to precisely match the electrode size. Ambient temperature during the measurement sessions was approximately 19 °C. For clarity, only the results for subject I are shown in the following plots.

TENS GEL that we use in experiments [27] is specifically designed for this purpose, does not contain formaldehyde and is free from salts, completely hypoallergenic, water-soluble, resistant to drying, odourless, and easily removable. It does not stain, does not grease, does not ionize and does not oxidize over time. It does not contain active ingredients that could damage the electrodes in any way. The manufacturer did not provide much information about the hydrogel used in our experiments; however, similar hydrogel materials and applications are discussed in [29].

## 5. Results

### 5.1. Measured Signals

**Figure 5:**
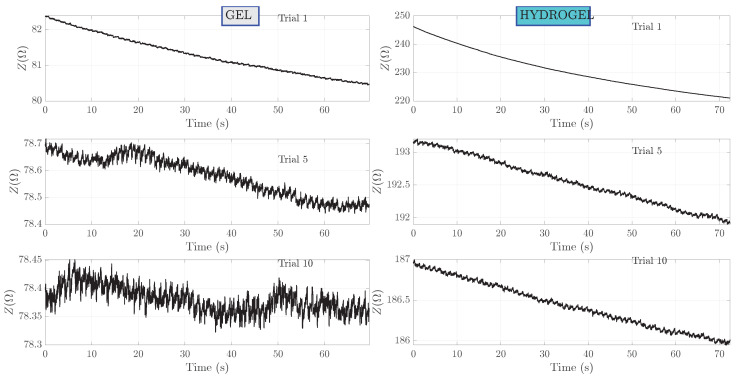
Raw bioimpedance measurements for subject I. Measurements no. 1, 5 and 10 are shown.

The recorded raw bioimpedance measurements are presented in Figure 5. Out of the 10 measurements, only three are presented—the first, the fifth and the last (10th) signal with the highest SNR, signal with the lowest SNR and signal obtained as an average of all 10 signals. These three signals are shown for both gel and hydrogel couplant cases. Bioimpedance values have a settling time—a trend to decrease over time and asymptotically tend to an equilibrium value. Bioimpedance starting values differ for different coupling agents. In the case of hydrogel, the starting bioimpedance value is about two to three times higher compared to measurements with gel. Moreover, the slopes of bioimpedance that decay over time are also different. In the case of gel coupling, the rate of decay is lower and small-amplitude oscillations can be seen for the fifth and tenth measurements. The signal obtained after placing the electrodes on the skin can be affected by the resulting capacitance of the skin layers. This can cause the signal to appear as a falling exponent, which can obscure the true nature of the signal and affect the accuracy of heart rate measurements, as mentioned in [30].

**Figure 6:**
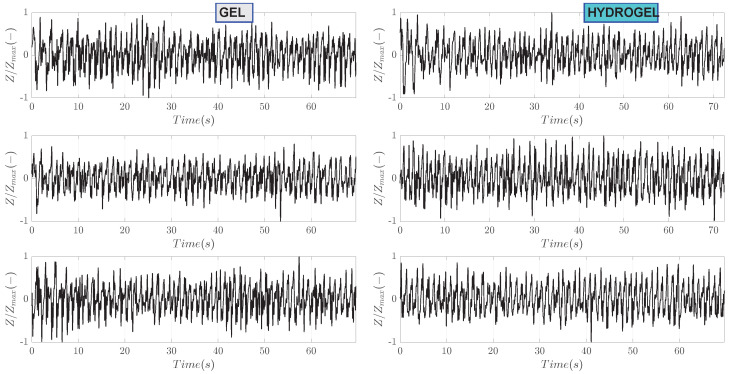
Band-pass filtered bioimpedance signals for subject I.

**Figure 7:**
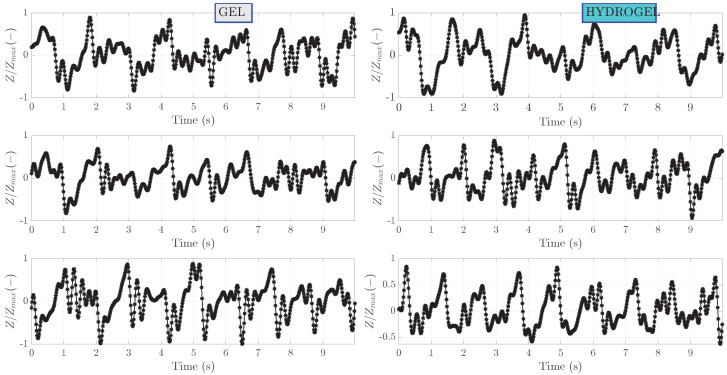
Zoomed-in on the first 10 s of band-pass filtered bioimpedance signals for subject I.

Bioimpedance signals processed with band-pass filtering are shown in Figure 6. This step has removed the slope of the raw signals as well as some noise outside the pass bands of the filter. For ease of comparison, these processed signals are also normalized to the range from −1 to 1. Periodic oscillations corresponding to heartbeat can be clearly seen. Zoom-in on the first 10 s of measurement is shown in Figure 7. A clear pattern of hemodynamic activity with the fiducial points can be seen.

**Figure 8:**
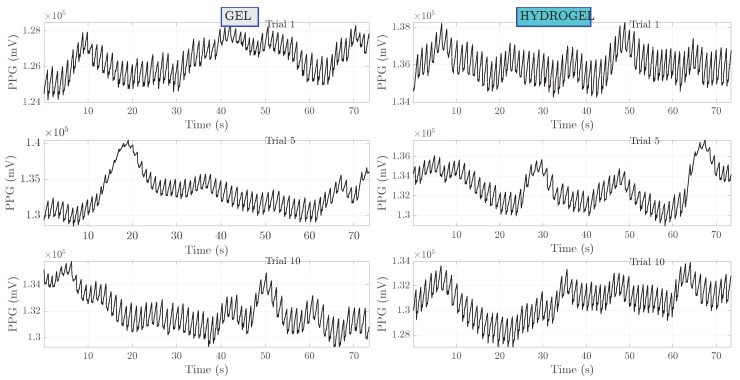
Raw PPG measurements for subject I. Measurements no. 1, 5 and 10 are shown.

**Figure 9:**
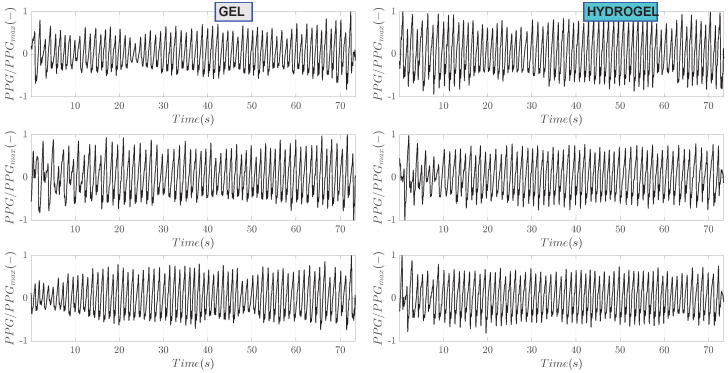
Band-pass filtered PPG signals for subject I. These signals correspond to the presented filtered bioimpedance signals.

**Figure 10:**
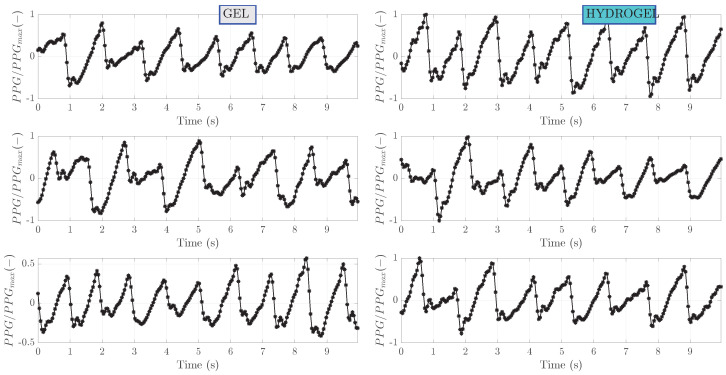
Zoom-in on the first 10 s of band-pass filtered PPG signals for subject I.

Results of raw PPG signals measured simultaneously with a bioimpedance are shown in Figure 8. These signals correspond to the ones presented for the bioimpedance. Several observations can be made. Firstly, compared to bioimpedance, the values of PPG do not change significantly with time. Secondly, there is a pronounced baseline wander. Thirdly, apart from higher frequency oscillations associated with a heart beat, smaller frequency components are presumably related to respiration. The respiration frequency is in the range 0.15–0.4 Hz [31]. Band-pass filtering of PPG signals, as shown in Figure 9 has removed a wander of baseline level and the zoom-in on the first 10 s is depicted in Figure 10.

### 5.2. Signal Stationarity and Normality

A more straightforward method of signal quality assessment in periodic signals is based on the autocorrelation function. This method involves computing the autocorrelation function of the signal. The SNR indicator is computed as $SNR=\frac{R(\tau =0)}{\frac{1}{N}\sum_{i=1}^{N}R\left(\tau_{i}\right)},$ where *R* is the correlation coefficient and τ are time lags of the signal with itself. The idea is that by combining the fact that, theoretically, the autocorrelation of noise is zero [32] and diminishing of autocorrelation values with increasing time lags, the mean over all time lags except at τ=0 corresponds to noise. Compared to the classical wavelet-based denoising method, this method requires less computational resources, since there is no need to decompose the signal into frequencies. However, the restriction of the autocorrelation method is that the signal has to be periodic, stationary and it should not be correlated with the noise.

The measured bioimpedance signals were tested regarding stationarity and normality in order to asses the appropriateness of the autocorrelation method for estimation of the SNR of bioimpedance signals. For this task, Kolmogorov–Smirnov, Anderson–Darling and Jarque–Bera statistical tests were conducted on the normalized bioimpedance signals to assess their normality. On the other hand, Augmented Dickey–Fuler test and Variance ratio test were carried out to test the stationarity of the recorded signals. Multiple tests were performed to increase the assurance of the judgment on the nature of the signals. The results on the signals measured on subject I are shown in Table 4 and Table 5. The tests were performed at a five percent significance level. All of the included signals are non-normal, since the associated p-value is less than α and statistic value is larger than the critical value. The same is true for non-stationarity tests. Hence, the measured bioimpedance signals are non-normal and non-stationary at α=0.05 and it is not correct to apply simpler signal quality estimation methods, such as the autocorrelation method.

### 5.3. Noise Detection

**Figure 11:**
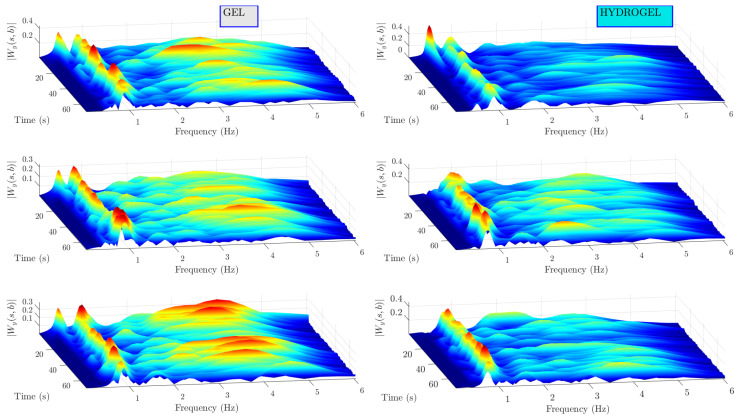
Bioimpedance scalograms for subject I. High amplitude can be traced around 1 Hz. Lower amplitude and lower duration signal components are present.

**Figure 12:**
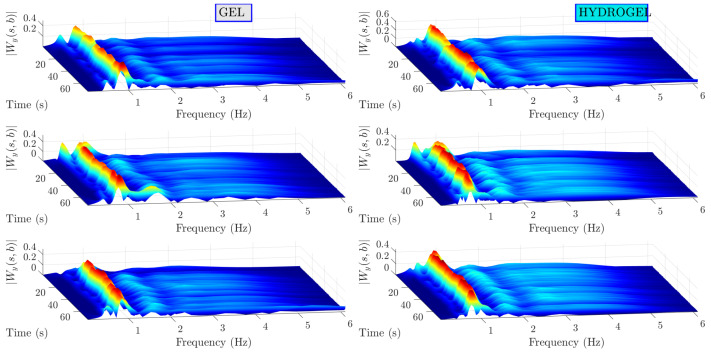
PPG scalograms for subject I. Dominating signal component is around 1 Hz.

Further, both band-pass-filtered bioimpedance and PPG measurements are processed with CWT. Scalograms of the bioimpedance signals are shown in Figure 11. A dominating wavelet ridge can be seen around 1 Hz on the frequency axis and can be traced for all time instants in all measurements conducted on subject I. It is clearly related to heart beat as the frequency if 1 Hz approximately matches the human heart beat at rest and the amplitude of modulus of CWT coefficients related to this signal component is much higher compared to other signal components. Relatively low amplitude CWT coefficients are observed at higher frequencies. Analogous results for PPG signals are illustrated in Figure 12. In this case, scalograms are dominated by a single signal component around 1Hz. These scalograms contain less noise compared to the ones of bioimpedance signals as is also evidenced by higher SNR indicator values.

**Figure 13:**
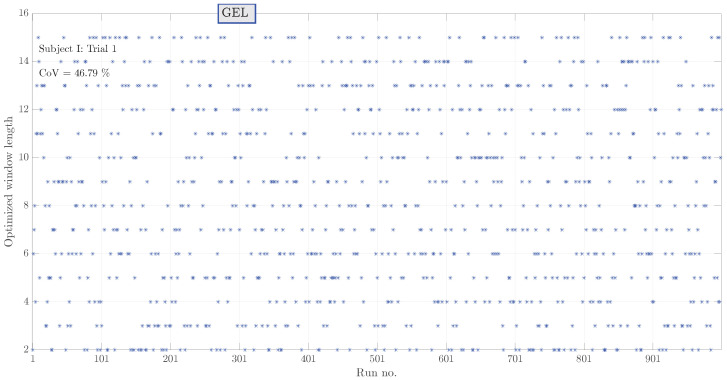
Values of the optimized window length values as obtained in 1000 runs of 10-fold cross-validation for subject I, trial 1. Each * denotes the result of an individual run. High coefficient of variation suggests that selection of a particular value is not critical.

Results of window length optimization for median filtering through 10-fold cross-validation are depicted in Figure 13. Here, window length values in a range from 1 to 15 were tested in a total of 1000 independent runs. A high scatter of values as indicated by the coefficient of variation indicates that no particular value is favorable over others. By increasing the number of runs from 100 to 1000, the coefficient of variation decreased by only approximately 1 percent. As there is no significance to a particular value of window length, it was decided to select the one that yields the maximum SNR.

**Figure 14:**
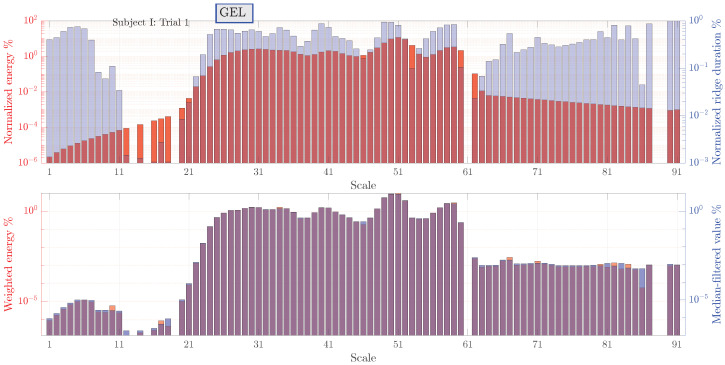
Normalized durations and energies of the ridges identified from the post-processed bioimpedance signals for subject I. Ridge durations normalized to a measurement length are shown as blue bars corresponding to the left *y* axis. Ridge energies normalized to the total signal energy are shown as red bars.

Median filtering results are illustrated in Figure 14. For clarity, these results are shown only for a trial I (gel) of subject I as intermediate results. The blue bars denote the scale parameters satisfying the ridge conditions. However, the duration of the associated ridges are not necessarily high. The red bars correspond to the relative energy expressed as percentage from the total energy of a signal. An overlay of both plots gives a visual indication of a mutual relationship between the ridge duration and its energy. The bottom plot shows weighted ridge energy (multiplication of both quantities from the upper plot). Hence, a ridge energy that is weighted by duration (red bars) is obtained. The maximum weighted energy is at a scale parameter of 51, which, in turn, corresponds to 0.868 Hz through scale-to-frequency conversion. Blue bars, on the other hand, are obtained from applying a median filter to the weighted energy data. A logarithmic scale is used so that the small and large amplitudes are clearly visualized.

### 5.4. Signal Quality

**Figure 15:**
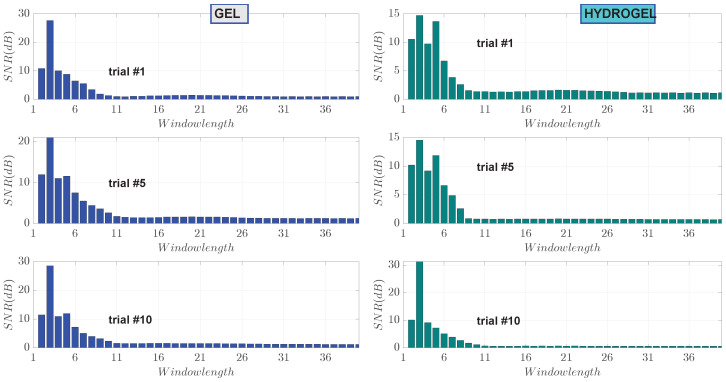
SNR values versus window length for the 1st, 5th and 10th trial measurements performed on subject I. The maximum SNR is consistently achieved at window length 3 both, for gel and hydrogel couplants.

The effect of window length of the median filter on estimation of the SNR was carried out. The SNR values versus window lengths ranging from 2 to 40 were calculated on measurements performed on subject I and shown in Figure 15. The first, fifth and tenth measurements are displayed both for gel and hydrogel couplants. It can be seen that the maximum SNR is consistently achieved at a window length of three. As the window length increases, the SNR values decrease and eventually stabilize where a further increase in window length does not have an impact on the SNR. In those cases, the weighted energy data are over-smoothed.

**Figure 16:**
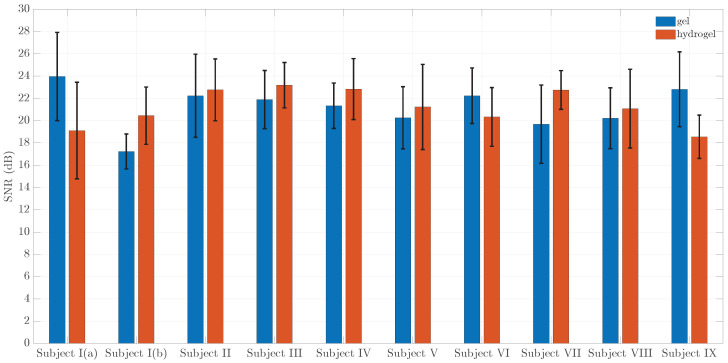
Mean SNR values for gel and hydrogel couplants for all test subjects. Error bars show confidence intervals.

The final results of the obtained SNR values are shown in Figure 16. Here, the mean values of the SNR are illustrated for all test subjects for both couplants along with vertical errorbars. The errorbars were calculated as confidence intervals $CI=t_{1-\alpha ,\nu }\times \frac{\sigma }{\sqrt{N}}$ and the final result can be presented as $SNR=\bar{SNR}\pm CI.$

The obtained mean SNR values range between 19 dB and about 24 dB. There are no significant differences in SNR results between gel and hydrogel couplants for all subjects—all differences are within the error bounds. Moreover, no significant differences are observed between test subjects. Hence, the choice of couplant has an impact mostly on practical aspects of measurement, such as availability, ease of application and personal comfort of the test subject rather than the actual quality of the measured signal. These bioparameters might show a correlation with specific features of the bioimpedance signals rather than the SNR values.

### 5.5. Classical Wavelet Transform Approach

The procedure of signal denoising using the classical wavelet transform approach is as follows. Signal decomposition into approximation and detail coefficients at different levels is performed using a discreet wavelet transform (DFT) with a selected mother wavelet function. The maximum level of decomposition depends on the length of the signal. A rule of thumb is to set it as equal or less than $\log_{2}\left(N\right),$ where *N* is length of the signal. The threshold level is calculated as

(11) T=TM × threshold_type 

where TM is the threshold multiplier, and “threshold_type” is the type of the threshold. Common choices include soft, hard or universal. The universal threshold is defined by $MAD\left(W_{x}\right)=\frac{median(|W_{x}-median\left(W_{x}\right)|)}{0.6745}.$ and MAD is median absolute deviation of wavelet decomposition coefficients $W_{x}$ of signal *x* representing a noise level in the signal.

A drawback of the classical wavelet transform approach for SNR estimation is that many parameters need to be provided by the user or found via an optimization process. These parameters include threshold type (soft, hard, universal or other), threshold multiplier and mother wavelet function. Even though the threshold type can be selected through trial and error, no particular guidelines exist for wavelet mother function and an appropriate value of threshold multiplier.

**Figure 17:**
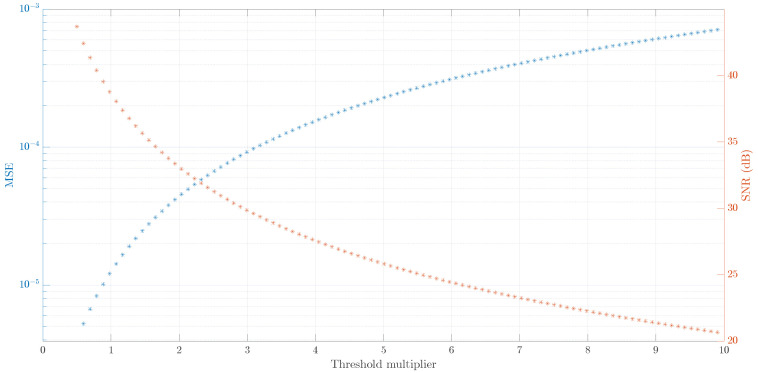
Estimation of threshold multiplier using classical wavelet transform method (subject I, trial 8, gel). Threshold multiplier is selected at the crossing of MSE and SNR curves. With this approach the SNR is equal to 33.4 dB.

In the current study, we attempt to present a solution to the selection of the threshold multiplier value. At each level of wavelet decomposition, a threshold *T* calculated for each value of TM is applied to wavelet transform coefficients to obtain a reconstructed denoised signal $x_{d}$. Then, a mean-squared error between the original signal and a reconstructed denoised signal is computed at each value of TM. Finally, the SNR is computed as

(12) $$SNR\left(TM\right)=10\times \log_{10}\frac{\left|\right|x_{d}\left(TM\right){\left|\right|}^{2}}{||x\left(TM\right)-x_{d}\left(TM\right){\left|\right|}^{2}}$$

where ||.|| is the norm. In this study, 100 linearly spaced threshold multiplier values ranging from 0.5 to 10 were tested to estimate an appropriate threshold multiplier. As a threshold multiplier increases, the threshold level increases as well, meaning more aggressive denoising as more wavelet decomposition coefficients are set to zero. As a consequence, the mean squared error (MSE) increases and the SNR decreases. This effect can be seen in Figure 17, where both of these quantities change monotonically. A breakpoint of TM is chosen where the MSE and SNR curves intersect. With this approach, SNR is equal to 33.4 dB and TM to 2.13. The disadvantage of this approach is that it is empirical as no strict guidelines exist on how to adequately choose TM.

## 6. Conclusions

The main goal of the study is the development of signal quality estimation technique based on continuous wavelet transform (CWT). This method requires identification of wavelet ridges, weighing their energy by a duration normalized to the duration of the whole signal and, lastly, performing median filtering of the weighted energies. Optimal median filtering is assured by selecting appropriate window length. The method is validated on nine test subjects, with different body mass indices, ages and levels of physical activity. The bioimpedance signals are measured with a wearable device fixed on a wrist. In addition, plethysmography (PPG) signals are measured from an index finger. We assess the effect of two different electrode couplants: gel and hydrogel.

In addition, this work also showcases a custom wearable device for human bio-impedance measurements. It has proven to be reliable according to the inspection of the measured signals. Nevertheless, it was necessary to carry out band-pass filtering to remove a negative slope and reveal the details of signal morphology.

The following findings must be highlighted:

1. Visualization of signal components in time-frequency plane aids in signal quality estimation. The most intensive signal components corresponding to heartbeat are traced through the whole duration of the signal. Other components, including noise, can also be traced in terms of their amplitude and duration as indicated by wavelet scalograms.
2. PPG signals are helpful to be correlated with bioimpedance signals to match the heartbeat component. PPG scalograms show that apart from the heartbeat component, signals are relatively clean. This means that the other components as seen in bioimpedance scalograms likely correspond to noise.
3. The measured bioimpedance signals are non-stationary and are not normally distributed, meaning that many existing methods for signal quality estimation such as autocorrelation are not applicable. The proposed method, on the other hand, is based on wavelet transform of the measured signal and, hence, can handle signal non-stationarity and non-normality.
4. The developed signal quality estimation method is fully adaptive. Compared to the classical wavelet-based signal denoising and signal-to-noise ratio estimation, there is no need to set fixed threshold levels or select proper threshold types. Instead, median filtering with an optimized window length is performed and noise is estimated from the available ridge information. SNR estimates obtained with the classical and the proposed methods are within error bounds.
5. The effect of coupling agent does not have a significant impact on signal quality as assessed by the SNR. All the differences are within the error bounds. Also, there is no evidence that physical activity levels, age and BMI of test subjects have a role in signal quality of the bioimpedance signals. Again, all differences are within error bounds.

The limitations of the applicability of the proposed SNR indicator include relatively high computational requirements. First, the signal needs to be pre-processed, as the low frequency slope had to be removed, since otherwise the energy of the slope’s frequency component might have been taken into account in calculation of the SNR. Secondly, computation of continuous wavelet transform with a desired resolution itself requires significant computational resources, especially for the median filtering via a cross-validation routine.

## Figures and Tables

**Table 1:** Qualitative comparison of different MCU and SoC. nRF5340 (bolded in the table) is selected for the wearable device.

Family	Name	Max Clock Frequency	Architecture	Radio
nRF52	nRF52840	64-MHz	Cortex M4F	BLE 5, IEEE 802.15.4, Propr. 2.4 GHz
nRF52	nRF52832	64-MHz	Cortex M4F	BLE 5.1, Propr. 2.4 GHz
**nRF53**	**nRF5340**	**128-MHz**	**Cortex M33**	**BLE 5.2, IEEE 802.15.4, Propr. 2.4 GHz**
TI SimpleLink	CC2652R	48-MHz	Cortex M4F	BLE 5.1, IEEE 802.15.4
MSP432	msp432p401r	48-MHz	Cortex M4F	No Built-in
STM32L	STM32L475	48-MHz	Cortex M4F	No Built-in
ESP32	ESP32	240-MHz	Xtensa LX6	BLE 5.0, WIFI, IEEE 802.11 b/g/n,
ESP32-C3	ESP32-C3	160-MHz	RISC-V	BLE 5.0, WIFI, IEEE 802.11 b/g/n,
EFR32	EFR32BG22	76.8-MHz	Cortex M33	BLE 5.2, 802.15.4 g
SAM3X	ATSAM3X8E	84-MHz	Cortex M3	No Built-in

**Table 2:** Performance comparison of different MCU and SoC. nRF5340 (bolded in the table) is selected for the wearable device.

Name	Active Current/Radio Current @0 dB					Avg. mA	RAM	FLASH
	mA	µA/MHz	Rx (mA)	Tx (mA)	RAM ret. (µA)	@ 48 MHz	kB	kB
nRF52840	3.328	52	4.6	4.8	3.16	53.1	256	1024
nRF52832	3.7	58	5.4	5.3	1.9	57.6	64/32	512/256
**nRF5340**	**7.3**	**57**	**3.8**	**4.2**	**2.3**	**57**	**512 + 64**	**1024 + 256**
CC2652R	3.4	70.8	6.9	7.3	0.94	69	80	352
msp432p401r	3.84	80	-	-	0.35	-	64	256
STM32L475	8	166.7	-	-	0.236	-	128	1000
ESP32	68	283.3	100	130	10	282.9	400	384
ESP32-C3	20	125	-	-	5	-	400	384
EFR32BG22	2.07	27	3.6	4.1	1.4	27.3	32	512
ATSAM3X8E	70.89	923	-	-	2.5	-	96	512

**Table 3:** Biological data of test subjects.

Subject	Gender	Age (Years)	Mass (kg)	Height (m)	BMI	Activity Level
I	male	34	76	1.80	23.46	high
II	male	54	94	1.84	27.76	low
III	male	22	57	1.78	17.99	medium
IV	male	30	75	1.95	19.72	medium
V	male	39	70	1.83	20.90	medium
VI	male	26	83	1.83	24.78	medium
VII	female	23	70	1.75	22.86	low
VIII	male	30	88	1.75	28.73	high
IX	male	24	105	2.03	25.48	low

**Table 4:** Normality test of bioimpedance data for subject I at α=0.05.

	Kolmogorov–Smirnov Test			Anderson–Darling Test			Jarque–Bera Test		
	*p*-Value	KS	KS Crit.	*p*-Value	AD	AD Crit.	*p*-Value	JB	JB Crit.
Gel #1	0	0.27	0.0197	0.0005	3.59	0.75	0.001	28.26	5.98
Gel #2	0	0.32	0.0195	0.0005	6.37	0.75	0.001	31.09	5.98
Gel #3	0	0.28	0.0197	0.0005	5.97	0.75	0.001	37.29	5.98
Gel #4	0	0.25	0.0196	0.0005	6.10	0.75	0.001	55.09	5.98
Gel #5	0	0.29	0.0197	0.0005	2.31	0.75	0.001	20.11	5.98
Gel #6	0	0.26	0.0196	0.0005	3.90	0.75	0.001	20.00	5.98
Gel #7	0	0.30	0.0193	0.0005	1.80	0.75	0.001	15.05	5.98
Gel #8	0	0.30	0.0203	0.0005	5.21	0.75	0.001	68.05	5.98
Gel #9	0	0.27	0.0198	0.0005	2.70	0.75	0.001	21.72	5.98
Gel #10	0	0.26	0.0198	0.0044	1.18	0.75	0.006	10.71	5.98
H.gel #1	0	0.36	0.0168	0.0005	2.29	0.75	0.001	240.36	5.98
H.gel #2	0	0.31	0.0191	0.0005	1.87	0.75	0.006	10.41	5.98
H.gel #3	0	0.27	0.0193	0.0005	5.52	0.75	0.003	12.57	5.98
H.gel #4	0	0.31	0.0196	0.0005	6.43	0.75	0.001	28.12	5.98
H.gel #5	0	0.26	0.0195	0.0005	4.17	0.75	0.001	37.50	5.98
H.gel #6	0	0.25	0.0196	0.0005	7.05	0.75	0.001	55.15	5.98
H.gel #7	0	0.26	0.0195	0.0005	15.19	0.75	0.001	89.20	5.98
H.gel #8	0	0.27	0.0194	0.0005	4.72	0.75	0.001	31.16	5.98
H.gel #9	0	0.26	0.0199	0.0005	7.07	0.75	0.001	41.09	5.98
H.gel #10	0	0.27	0.0195	0.0005	12.84	0.75	0.001	81.95	5.98

**Table 5:** Stationarity test of bioimpedance data for subject I at α=0.05.

	Augmented Dickey–Fuller Test			Variance Ratio Test		
	*p*-Value	ADF	ADF Crit.	*p*-Value	VR	VR Crit.
Gel #1	0.001	−6.79	−1.94	0	35.14	1.96
Gel #2	0.001	−7.51	−1.94	0	34.97	1.96
Gel #3	0.001	−6.88	−1.94	0	35.26	1.96
Gel #4	0.001	−7.43	−1.94	0	36.39	1.96
Gel #5	0.001	−7.06	−1.94	0	33.30	1.96
Gel #6	0.001	−7.26	−1.94	0	34.65	1.96
Gel #7	0.001	−7.53	−1.94	0	36.12	1.96
Gel #8	0.001	−7.43	−1.94	0	34.97	1.96
Gel #9	0.001	−7.23	−1.94	0	33.79	1.96
Gel #10	0.001	−7.68	−1.94	0	34.22	1.96
Hydrogel #1	0.001	−7.76	−1.94	0	40.81	1.96
Hydrogel #2	0.001	−6.55	−1.94	0	35.91	1.96
Hydrogel #3	0.001	−6.44	−1.94	0	33.95	1.96
Hydrogel #4	0.001	−6.11	−1.94	0	34.35	1.96
Hydrogel #5	0.001	−6.92	−1.94	0	34.74	1.96
Hydrogel #6	0.001	−6.18	−1.94	0	35.13	1.96
Hydrogel #7	0.001	−6.86	−1.94	0	36.19	1.96
Hydrogel #8	0.001	−6.76	−1.94	0	33.42	1.96
Hydrogel #9	0.001	−5.97	−1.94	0	34.17	1.96
Hydrogel #10	0.001	−6.34	−1.94	0	34.92	1.96

## Data Availability

Data available upon request.
